# Supplementary material for: Identification of photoperiod-induced specific miRNAs in the adrenal glands of Sunite sheep (Ovis aries)
Source: Front Vet Sci. 2022 Jul 22;9:888207. doi: 10.3389/fvets.2022.888207 (PMC9354845; doi:10.3389/fvets.2022.888207)
Supplement: Supplementary file 1 [file Data_Sheet_1.ZIP › Supplementary file/miRDeep2 file.zip!/miRDeep2 file/Novel_Structure/LP42A3/Novel_105.pdf]

Provisional ID	:	NC_019463.2_9294
Score total	:	2.2
Score for star read(s)	:	-1.3
Score for read counts	:	0
Score for mfe	:	1.9
Score for randfold	:	1.6
Score for cons. seed	:	
Total read count	:	144
Mature read count	:	144
Loop read count	:	0
Star read count	:	0

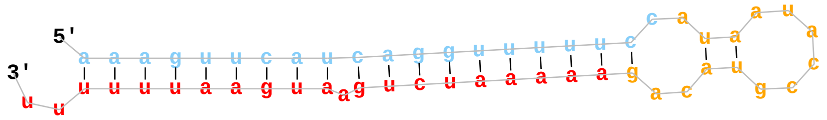

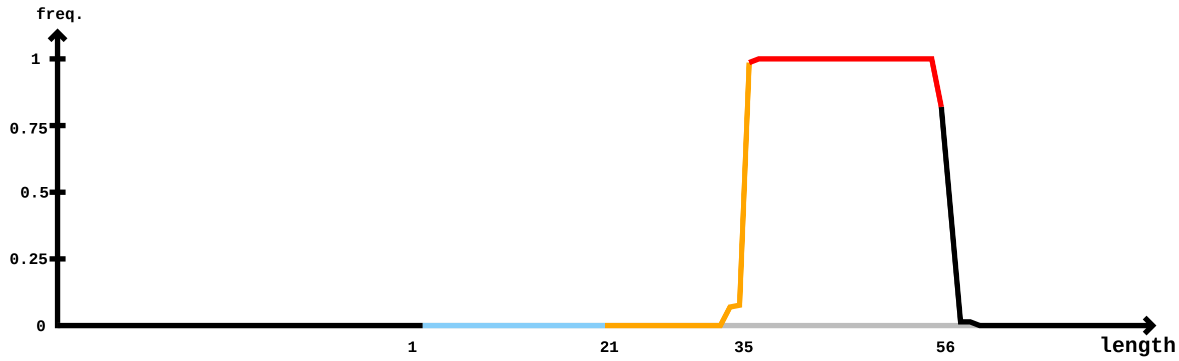


```
                                        Star                          Mature
5'- ccagguagagggaauaguacguauugauuuggccagaaaguucaucagguuuuuccauaauaccguacagaaaaaucugaaugaauuuuuuggccaauccaguacaaaggug   -3'    exp
    ((........))........((((((..(((((((((((((((((((((((((((..((......))..)))))))))).)))))))))))))))))..)))))).......    reads   mm   sample
    ....................................................................agaaaaaucugaaugaaCuuuu......................    2       1    seq
    ....................................................................agaaaaaCcugaaugaauuuuu......................    1       1    seq
    ....................................................................agaaaaaucugaaugaaCuuuuu.....................    7       1    seq
    .....................................................................gaaaaaucugaaugaaCuuuuu.....................    1       1    seq
    ......................................................................aaaaaucugaaugaaCuuuu......................    23      1    seq
    ......................................................................aaaaaucugaaugaaCuuuuu.....................    65      1    seq
    ......................................................................aaaaaucugaaugaauuuuuug....................    1       0    seq
    ......................................................................aaaaaucugaaugaaCuuuuug....................    42      1    seq
    .......................................................................aaaaucugaaugaaCuuuuuggc..................    2       1    seq
```
